# Nursing students’ perceptions and experiences of student–lecturer evaluation as a strategy for improving teaching and learning at the University of Namibia

**DOI:** 10.4102/curationis.v49i1.2875

**Published:** 2026-08-31

**Authors:** Ritu K. Stanislaus, Daniel O. Ashipala, Julia Amadhila

**Affiliations:** 1 Department of General Nursing Sciences, Faculty of Health Sciences and Veterinary Medicine, University of Namibia, Rundu, Namibia

## Abstract

**Background:**

In higher education, the student–lecturer evaluation (SLE) system is a critical mechanism for enhancing teaching quality and fostering student-centred learning. Despite its importance, limited research exists in Namibia on students’ perceptions and experiences of this evaluation process, particularly regarding its effectiveness in improving teaching and learning outcomes.

**Objectives:**

The study aimed to explore and describe nursing students’ perceptions and experiences regarding the SLE system at the Rundu Campus of the University of Namibia (UNAM).

**Method:**

A qualitative, explorative, descriptive and contextual design was employed. Fifteen students were purposively selected to participate in the study. Data were collected through semi-structured interviews, which were audio-recorded and transcribed verbatim. Thematic analysis was used to analyse the data and identify emerging themes and sub-themes.

**Results:**

Three main themes and nine subthemes were identified from the data analysis. This study explored nursing students’ perceptions and experiences regarding the use of SLE as a strategy for improving teaching and learning at the UNAM. Participants highlighted both the benefits and challenges of SLE, including its potential to improve teaching quality, as well as barriers such as lack of feedback, fear of reprisal, poor communication, late notifications and limited transparency.

**Conclusion:**

The study concluded that while SLEs have significant potential to improve teaching and learning, their effectiveness is constrained by administrative, logistical and communication challenges. Addressing these barriers and implementing students’ recommendations can enhance the credibility, accessibility and impact of evaluation systems.

**Contribution:**

The findings provide valuable insights for universities to develop ongoing strategies and targeted interventions to improve the implementation of SLEs, foster accountability and promote continuous enhancement of teaching and learning quality.

**Keywords:**

experiences; feedback; higher education; Namibia; perceptions; student–lecturer evaluation; teaching quality.

## Introduction

Institutions of higher education use student–lecturer evaluation (SLE) systems to rate the education provided to students. Essentially, these evaluations are widely used by such institutions to measure teaching quality. In addition, SLE is used by lecturers themselves to reflect on their own teaching practices with a view to improving instructional effectiveness (Talvio & Vuorinen 2026); SLEs have become an important topic in recent decades. Through this process, lecturers receive feedback on their teaching from students, which supports reflection and improvement by strengthening teaching competencies and enhancing teaching effectiveness (Song & Lee 2020). Therefore, university teaching and learning evaluation systems are designed primarily to help lecturers align their intended instructional messages with how these messages are perceived by students.

Beyond measuring teaching quality and providing feedback, SLE allows lecturers to reflect on their teaching and, fundamentally, helps recognise their essential role in university learning and teaching processes. Feedback from students is significant because it highlights the effectiveness of teaching and learning (Song & Lee 2020). Many universities worldwide have developed various forms of SLE in a bid to improve the quality of teaching and learning with a view to improving student performance (Arnaiz-Sánchez et al. 2020; Contrino et al. 2024).

Effective use of student feedback is highly motivating for both parties: It not only provides valuable information to students but also enables lecturers to inform and shape their teaching practices. Course and student lecturer evaluations reflect qualities associated with good teaching such as lecturers’ knowledge, clarity, classroom management and course organisation (Constantinou & Wijnen-Meijer 2022). It is essential for academic institutions to be aware of students’ opinions of their lecturers, as it also provides an opportunity to identify students’ needs (Mngomezulu 2025).

Over the last decade, awareness of the significance of quality assurance in academic institutions has grown worldwide, as evidenced by developments in Israel, Western Europe and the United States (Atanaw, Estifanos & Negash 2025). This heightened awareness is a result of both the considerable competition among institutions and the increasing diversity of the student population (Chigbu & Makapela 2025; Jain et al. 2025; Sabharwal 2020). Student–lecturer evaluation also contributes to professionalism by enabling lecturers to identify potential teaching improvements (Samonova et al. 2025) and, consequently, improving students’ attainment of learning outcomes (Makhijani & Dugaje 2025). Internationally, universities have increasingly adopted various forms of SLE to improve the quality of teaching and enhance overall student performance (Bernhard et al. 2024). For instance, Siziba (2022) noted that Great Zimbabwe University employs online SLE specifically designed to enrich the lecturing and learning process.

Much of the research on SLEs has focused on their validity (Quansah et al. 2024), which is particularly important when they are used to assess and measure lecturers’ professional performance. Extant research has indicated a number of areas of potential bias in SLEs, which are linked to various factors, including the course (for example subject disciplines, higher academic levels, course difficulty and compulsory status); the lecturer (for example the ‘halo’ effect of charismatic lecturers, gender, race, sexual orientation and rank, with professors often rated more highly); the university (for example class size and timetabling); and the students (for example, maturity, gender and grade expectations) (Kim, Johnston & Fan 2024; Lavidas et al. 2022).

According to the Ministry of Education (2007), Namibia has introduced several key policies to enhance educational quality, including the National Standards and Performance Indicators for Schools (NSPI), the National Professional Standards for Teachers in Namibia and the Education and Training Sector Improvement Programme (ETSIP). The NSPI policy, introduced in 2005, served as a guide for school assessors to evaluate school performance (Ministry of Education 2007). Despite these efforts, it was revealed that only a few institutions had established SLE as a tool for measuring effective teaching (Ministry of Education 2007).

The University of Namibia (UNAM) policy on SLE states that all lecturers are required to be evaluated by their students according to the modules they teach. These evaluations are administered once each semester to assess lecturers’ instructional practices and students’ learning experiences. Currently, all evaluations of learning and teaching are managed through an online platform known as the Education Surveys Automated Suite (EvaSys). Education Surveys Automated Suite is a proprietary, automated education survey platform housed within the Learning and Teaching Enhancement section in the University’s Centre for Innovation in Teaching and Learning, where students complete an online questionnaire to evaluate their lecturers and modules. However, in a move towards greater institutional efficiency and reduced expenditure on software licensing, the UNAM is planning to relocate all evaluation functions to the Moodle learning management system, an open-source and freely available platform. Fundamentally, the information derived from the SLE process is used strategically by the University to inform evidence-based decision-making, ultimately leading to the improvement of teaching and learning across the institution.

The UNAM employs SLEs as a mechanism to assess and enhance the quality and effectiveness of teaching. While the literature generally supports the notion of these evaluations contributing to academic development and teaching enhancement (Constantinou & Wijnen-Meijer 2022; Crimmins et al. 2025; Sidwell et al. 2025), the practical application and impact of these evaluations at the UNAM, particularly at the Rundu campus, remain unclear. Specifically, despite the institution-wide implementation of SLEs, there is a significant gap in understanding the perceptions and experiences of nursing students regarding the use of these evaluations as a strategy to improve teaching and learning at the Rundu Campus. This lack of focused research leaves crucial questions unanswered about the effectiveness and impact of this evaluation strategy from the perspective of nursing students within this localised educational setting.

## Research methods and design

### Study design

A qualitative, explorative, descriptive and contextual design was employed. This design was used to explore and describe nursing students’ perceptions of the use of SLEs as a strategy to improve teaching and learning at the UNAM. According to Maree (2016), qualitative research design is naturalistic in nature, focusing on natural settings where interactions occur. In addition, it seeks to explore how people make sense of their surroundings, experiences and understanding of phenomena. Therefore, this design was suitable for exploring the perceptions and experiences of nursing students who had spent more than a year at the UNAM regarding the use of SLEs as a strategy to improve teaching.

### Study population and sampling strategy

The study population consisted of undergraduate nursing students who had spent more than a year at the UNAM. According to records from its Rundu campus, the total population of undergraduate students is 350. This constituted the population for this study. Specifically, the population comprised all second-, third- and fourth-year undergraduate nursing students at the UNAM. Convenience sampling was employed to select participants from the nursing student population. This non-probability sampling method was chosen because it relies on the participants’ ready availability and accessibility to the researcher at the time of data collection. Accordingly, second-, third- and fourth-year nursing students who were readily available and willing to participate were selected after providing informed consent at the Rundu Campus during the designated data collection period. The selection of these academic levels was based purely on the following inclusion criteria, namely: (1) Undergraduate nursing students at the UNAM Rundu campus who have been studying for more than a year, and (2) undergraduate nursing students at the UNAM Rundu campus willing to participate. This study’s exclusion criteria were the following, namely: (1) Nursing students in the first year of study, as they have limited exposure to SLEs, (2) any second-, third- and fourth-year nursing students who were not willing to participate and (3) any second-, third- and fourth-year Nursing students who were willing to participate but were not available at the time of data collection. Data collection continued until data saturation was reached with 15 participants, after which two additional participants were included to confirm the consistency of the information.

### Data collection

This study was conducted between June and September 2025. The primary data collection instrument was a semi-structured interview guide developed based on the research objectives, research questions and relevant literature. Data were collected through face-to-face semi-structured interviews guided by this interview guide, and all interviews were recorded with the participants’ permission. Face-to-face interviews allowed the researcher to observe non-verbal communication and enabled both the interviewer and participants to seek necessary clarification. Although qualitative research often employs multiple data collection tools, this study primarily used semi-structured interviews. Data for the study were collected through these face-to-face interviews, and the research questions used during the interviews included the following: (1) *Please tell me your personal understanding of the concept of student lecturer evaluation?* (2) *Please tell me what are your perceptions of using a student’s lecturer evaluation as a strategy to improve teaching and learning at the UNAM?* (3) *Please tell me what are your experiences of using a student’s lecturer evaluation as a strategy to improve teaching and learning at the UNAM?* (4) *What strategies can be adopted to improve the quality of teaching and learning at the UNAM by the use of student lecturer evaluation?*

### Data analysis

Raw data from the audio-recordings were transcribed verbatim before they were analysed using Braun and Clarke’s (2019) six steps of data analysis: (1) The researcher familiarised himself with the data by rereading interview transcripts, listening to the recordings multiple times and taking notes from the data. (2) Then he generated the initial codes in order to describe the content and identify and provide a label for features of the data that were relevant to the research question. The researcher coded and transcribed the data himself as he was more familiar with the study objectives and questions. By utilising the Microsoft document, in vivo codes were generated through a meticulous process of reading each word and line and then highlighting relevant portions of the data using distinct colours. Additionally, the researcher employed concise labels to enhance clarity when referring to specific codes within all the transcripts. Subsequently, the researcher systematically searched, sorted, and compiled all related codes into potential themes on the basis of students’ thoughts, views, understanding and interpretations of their experiences and perceptions towards SLE. (3) Searching for themes in the data of all the interview transcripts by reviewing the coded data to identify areas of similarities and overlap. (4) Reviewing potential themes by checking them against the organised extracts of the data and if the themes work in relation to the data. (5) Defining and naming themes by summarising each theme in a few sentences to produce a coherent and overall account of the data. Themes were then reviewed for relevance and clarity and were tested for referential adequacy by returning to the raw data. The themes were then defined and named before succinct themes identifying larger themes were written as unique stories. An independent coder, holding a Doctorate in Nursing Education and specialising in qualitative research, conducted an autonomous thematic analysis of the data. The findings were then cross-referenced with the primary researcher’s analysis to ensure validation. Units of significance were classified and subcategorised based on client viewpoints, ultimately leading to the identification of themes. Participants were uniquely coded from P1 to P15, and pseudonyms for face-to-face interviews were created, followed by a number. (6) Producing a report by writing a clear, convincing and complex description of the data based on the data analysis. The researcher and an independent coder identified and agreed on the main themes.

### Trustworthiness of the study

Criteria outlined by Lincoln and Guba (1985), which are widely accepted by qualitative researchers and form the focus of this section, were used to ensure the rigour of the study. These criteria include credibility, dependability, conformability and transferability.

In this study, credibility was ensured through three strategies. Firstly, triangulation was applied by gathering data from multiple sources, including interviews, observations and field notes, to obtain a comprehensive understanding of nursing students’ experiences. Secondly, member checking was undertaken where the researcher shared the preliminary findings, interpretations and conclusions with the participants to verify their accuracy and ensure that their accounts truly reflected their experiences. After the initial data collection and analysis, the researcher returned to the nursing students to present the findings derived from their data. Participants were asked to review the findings and provide feedback on whether they accurately represented their experiences or perceptions. Clarification, additional insights, or corrections were sought based on participants’ feedback, thereby enhancing the trustworthiness of the interpretations.

Thirdly, persistent observation was used to gain an in-depth understanding of the phenomenon. This involved focusing on the most relevant and salient aspects of nursing students’ perceptions and understanding of SLE as a strategy for improving teaching and learning. By dedicating sufficient time and attention to these core elements, the researcher was able to identify key patterns and recurring issues, adding depth and detail to the findings.

Dependability was ensured through a detailed and transparent description of the research methodology, including the data collection process, interview guide and data analysis techniques. All the data generated during the study were accurately recorded, and interview transcripts were kept available to verify interpretations and findings. In addition, the findings were systematically compared with and supported by relevant and established literature on SLE as a strategy for improving teaching and learning at the UNAM, thereby enhancing the consistency and dependability of the findings.

Confirmability was ensured through the use of techniques such as inquiry audits, reflexivity and triangulation. Inquiry audits involved engaging an external expert to examine the collected data and determine whether they adequately supported the investigation. Reflexivity, as described by Creswell (2014), required the researcher to reflect on how their personal background, beliefs and views may have influenced the study. Triangulation, defined as the use of more than one method to answer a research question (Brink, Van de Walt & Van Rensburg 2018), was further strengthened through the use of literature review findings, voice recordings and field notes, which were maintained as part of the audit trail. These measures contributed to both credibility and conformability.

Transferability was ensured through the use of thick description. This was achieved by providing comprehensive details of the study methodology, including the research setting, participant demographics, sample size and selection process, as well as the inclusion and exclusion criteria.

### Ethical considerations

Ethical clearance to conduct this study was obtained from the UNAM School of Nursing Public Health Ethical Committee (No. SoNPHEC 11/2025) on 23 April 2025 and the Ministry of Health and Social Services Institutional Review Board (No. 22/4/2/3) on 08 May 2025. Following approval, permission to participate in the study was obtained from all willing participants through the signing of an informed consent form.

Participants’ right to withdraw from the study at any time without penalty was respected, and they were informed of their right to seek clarification whenever necessary. The information of participants who took part in the study was kept anonymous and confidential by storing it on a password-protected computer accessible only to the researcher. Confidentiality was ensured throughout the study by safeguarding all participant information, and anonymity was maintained through the use of pseudonyms. No participant’s name was linked to any data or disclosed in any form.

## Results

### Description of study participants

The study involved a total of 15 nursing students from the UNAM Rundu Campus. As shown in the Table 1, the participants consisted of both male and female students from various years of study. The majority of participants were in their fourth year, with a few from the second and third years. The participants’ ages ranged from 22 to 26 years, indicating that most were young adults, typical of undergraduate university students. Female participants (eight) slightly outnumbered male participants (seven), showing a fairly balanced gender representation. Table 1 presents the demographic characteristics of the participants.

**TABLE 1:** Demographic characteristics of the participants.

Participant number	Age (years)	Gender	Year of study
P1	22	Female	Fourth year
P2	24	Female	Fourth year
P3	26	Male	Fourth year
P4	23	Male	Second year
P5	25	Female	Fourth year
P6	23	Male	Fourth year
P7	24	Male	Fourth year
P8	22	Female	Fourth year
P9	26	Male	Fourth year
P10	23	Female	Second year
P11	25	Male	Fourth year
P12	24	Female	Third year
P13	22	Male	Third year
P14	24	Female	Third year
P15	23	Male	Third year

### Presentation of the findings

Three main themes and nine subthemes were identified from the data analysis. This study explored nursing students’ perceptions and experiences regarding the use of SLE as a strategy for improving teaching and learning at the UNAM. The themes reflected students’ understanding of the purpose and value of SLE, factors influencing participation and engagement, and the need for institutional accountability and improvement of the evaluation system.

The themes and subthemes are presented in Table 2.

**TABLE 2:** Themes and subthemes emerging from the data analysis.

Main theme	Subtheme	Central meaning	Supporting quotation
Theme 1: Understanding the purpose and value of SLE	1.1. Perceptions of the evaluation process	Students viewed SLE as a formal feedback mechanism used to assess lecturers’ teaching effectiveness and professional conduct.	‘Student–lecturer evaluation is a process whereby students provide feedback on the teaching and effectiveness of the lecturers.’ (P2)
	1.2. Evaluation as a tool for improving teaching and learning	Students believed evaluations could improve teaching quality, identify strengths and weaknesses, and enhance learning outcomes.	‘This feedback can help lecturers identify areas of strength and weakness.’ (P6)
	1.3. Perceived relevance and educational value	Students regarded SLE as important because it gives them a voice in improving educational quality and learning experiences.	‘It’s important because it helps improve how lecturers teach.’ (P15)
Theme 2: Factors influencing student participation and engagement	2.1. Motivation and willingness to participate	Participation depended on whether students believed their opinions would be valued and acted upon.	‘Most students are willing to participate if they know their opinions matter.’ (P3)
	2.2. Fear, mistrust and lack of transparency	Fear of identification, absence of feedback and lack of visible institutional action discouraged honest participation.	‘Some fear being identified despite anonymity.’ (P5)
	2.3. Practical and communication barriers	Poor timing, inadequate reminders, and technological challenges limited meaningful participation.	‘We receive the links at the end of the semester.’ (P3)
Theme 3: Institutional accountability and strengthening of the evaluation system	3.1. Need for institutional accountability and follow-up	Students expected universities to demonstrate accountability by acting on evaluation outcomes and communicating results.	‘We never get feedback on the results of our evaluation.’ (P15)
	3.2. Improving accessibility and implementation systems	Students recommended mobile-friendly platforms, improved scheduling and better logistical planning.	‘The online system should be made mobile-friendly.’ (P14)
	3.3. Enhancing awareness and student engagement strategies	Students emphasised orientation, reminders and awareness campaigns to strengthen participation and understanding.	‘It would help if the university included a proper session about the evaluation during orientation.’ (P3)

SLE, student–lecturer evaluation.

### Theme 1: Understanding the purpose and value of student–lecturer evaluation

This theme encapsulates students’ conceptions about SLE and the significance they ascribed to the procedure in the classroom. Participants described evaluation as a tool for evaluating teaching efficacy, enhancing educational quality and boosting students’ engagement in academic processes rather than just an administrative task. Students’ greater awareness of evaluation as a tool for quality assurance and a forum for student opinion is reflected in the theme.

#### Subtheme 1.1: Perceptions of the evaluation process

The majority of participants perceived SLE as a formal, planned process that allows students to give feedback on lecturers’ classroom engagement, professional behaviour and teaching effectiveness. Students characterised the evaluation process as a chance to share their thoughts about the effectiveness of lecturers and the quality of their instruction. One participant explained:

‘My clearest understanding of this whole student–lecturer evaluation thing is that it is supposed to be a formal and really important way for us to give feedback; it’s basically a dedicated platform where we can tell the university about the teaching quality and how effective the lecturers have been all semester long.’ (P2)

Another participant added:

‘What I think evaluation means is that it’s a tool specifically designed to let us, the students, give our honest views on the lecturer’s performance in the classroom.’ (P10)

#### Subtheme 1.2: Evaluation as a tool for improving teaching and learning

Students believed that lecturer evaluations were intended to highlight both areas of strength and areas in need of development, ultimately leading to improved academic achievement. Because they thought the feedback could help lecturers reflect on their teaching strategies, identify areas of weakness and adopt more effective approaches to classroom instruction, participants strongly associated SLE with continuous progress in teaching and learning. One participant stated:

‘The main reason for doing this, in my opinion, is definitely to make the quality of teaching and learning much better across the whole nursing course.’ (P15)

Similarly, another participant reported:

‘We understand that this evaluation is meant to be a tool for improving things; it’s used to help the lecturers figure out what parts of their teaching style are working well.’ (P12)

#### Subtheme 1.3: Perceived relevance and educational value

Students highlighted the importance of SLE in offering chances for student participation in educational decision-making. Because it gave them the opportunity to voice concerns about how instructional strategies impacted their learning experiences, participants saw the evaluation process as empowering. Additionally, students felt that SLE provided a forum for them to voice their thoughts in order to enhance instruction, build stronger bonds between instructors and students, and foster a more encouraging learning atmosphere. As a result, the evaluation procedure was seen as pertinent and advantageous to students’ academic achievement and general educational experience. A participant explained:

‘I think the evaluation is extremely important and truly relevant because it really helps make improvements in how our lecturers teach.’ (P5)

Another participant commented:

‘The evaluation system is relevant because it is a quick way for us to point out problems in the teaching that could be hurting our grades.’ (P14)

### Theme 2: Factors influencing student participation and engagement

This subject explains the practical, emotional and institutional elements that influenced students’ involvement in SLE. Students’ desire to participate was heavily impacted by their judgments of institutional responsiveness, their trust in the system, and the practical aspects of the evaluation process, even though they largely acknowledged the significance of assessments.

#### Subtheme 2.1: Motivation and willingness to participate

Students were more inclined to participate in the evaluation process when they felt their opinions were valued and could lead to improvements in teaching and learning. Meaningful participation was strongly associated with perceptions of institutional responsiveness and respect for student opinions. One participant stated:

‘Most of the students are very ready to take part in the lecturer evaluation if they are convinced that their honest opinions will actually matter.’ (P3)

However, some participants felt discouraged when evaluations appeared to have little impact:

‘Students will just ignore the evaluation because they truly believe that the whole thing has no real impact.’ (P6)

#### Subtheme 2.2: Fear, mistrust and lack of transparency

Fear of being identified and ambiguity about how input was handled were proven to greatly hinder honest engagement. Numerous students voiced concerns about potential victimisation or unfavourable outcomes from instructors despite assurances of confidentiality. Additionally, several students were reluctant to give candid and thorough input during the evaluation process because they thought that the lack of transparency surrounding evaluation findings diminished their trust in the system. One participant explained:

‘I know many students who are often scared of being found out and then maybe punished later by the lecturers.’ (P5)

Another participant highlighted the absence of transparency:

‘We seriously never get any information about the results or summaries of our evaluation forms.’ (P5)

#### Subtheme 2.3: Practical and communication barriers

Inadequate time, few reminders and technical difficulties were among the operational challenges that students cited as impeding their participation. Many participants reported that they were unable to provide thoughtful answers when they received evaluation links during exam periods. Inadequate communication and network issues further decreased involvement and had an impact on the calibre of student responses. One participant noted:

‘The biggest issue we have with the timing is that we almost always get the links for the evaluation right when the semester is ending.’ (P3)

Another participant added:

‘A lot of students actually miss the deadline because the university sometimes fails to properly send out notifications or reminders early enough.’ (P6)

### Theme 3: Institutional accountability and strengthening of the evaluation system

Students’ suggestions for enhancing the legitimacy, efficacy and sustainability of SLE inside the university are reflected in this theme. Participants emphasised the significance of student awareness, accountability, transparency and accessibility in bolstering the evaluation process.

#### Subtheme 3.1: Need for institutional accountability and follow-up

Participants emphasised that assessments would only have significance if colleges showed accountability by sharing results and reacting to student input. When there was no obvious action or follow-up following evaluations, students became disheartened and began to question the process’ efficacy. Participants thought that disclosing evaluation findings and making changes would boost confidence, promote truthful involvement and bolster the evaluation system’s legitimacy. One participant stated:

‘The university policy needs to clearly state that the evaluation results should actually affect the lecturers’ jobs.’ (P11)

Another participant explained:

‘For the evaluation to actually be useful, the university definitely must share the results and key findings.’ (P8)

#### Subtheme 3.2: Improving accessibility and implementation systems

To promote broader student participation, participants recommended improving the organisation and technical accessibility of the review process. Students stressed that alternate solutions and mobile-friendly methods were particularly crucial for people who were having trouble connecting. Additionally, participants thought that enhancing the evaluation process’s administration, scheduling, and accessibility would lower technical obstacles and facilitate students’ ability to give insightful comments. One participant stated:

‘The online system should definitely be made mobile-friendly so we can easily fill it out using our phones.’ (P4)

Another participant added:

‘The university should try to provide offline options for students struggling with poor connectivity.’ (P6)

#### Subtheme 3.3: Enhancing awareness and student engagement strategies

Students recommended raising knowledge of the goal of SLE and enhancing communication. They believed that frequent orientation sessions and reminders would boost involvement and enhance the calibre of feedback. Additionally, participants stressed the need for ongoing support and more precise explanations of how SLE enhances instruction and learning. One participant commented:

‘It would be very helpful if the university could include a proper session about the evaluation during student orientation.’ (P3)

Another participant added:

‘The school needs to send out clear reminders at least two weeks before the evaluation closes.’ (P5)

## Discussion

The findings revealed that students largely perceive the SLE system as an institutional tool aimed at improving teaching quality and enhancing accountability. Students understood that the purpose of the evaluation is to assess lecturer performance, teaching methods, communication, professionalism and course delivery. This perception aligns with Donaldson (2020), who argues that student evaluations are essential mechanisms for monitoring academic standards and ensuring quality education.

Students also perceived the evaluation as a platform to express concerns anonymously, reinforcing findings by Zhao et al. (2022), who state that anonymity encourages honesty, especially in higher education settings. However, some students viewed the SLE as merely procedural, with limited impact on actual teaching practices. This contrasts with Constantinou and Wijnen-Meijer (2022), whose study indicated that in institutions where evaluations are taken seriously, students see clear improvements in teaching strategies. In addition, some students perceived the evaluation items as unclear or not fully reflective of their learning experiences. This supports Alsahil, Abdel Latif and Alsuhaibani (2024), who found that overly generic or poorly contextualised evaluation tools limit accuracy and engagement. Overall, while perceptions were generally positive regarding purpose, many students questioned the usefulness and responsiveness of the system.

A few students reported positive experiences, noting that the evaluation provided an opportunity to share their views without fear of identification. This aligns with Sultan et al. (2021), who found that students are more willing to offer constructive feedback when anonymity is guaranteed. Students also appreciated that the evaluation could be done online, increasing convenience and accessibility. According to Zhou and Kim (2024), digital systems enhance participation by reducing logistical barriers and allowing students to complete evaluations at their own pace. Some students indicated that the SLE system demonstrated the institution’s intention to improve academic quality. This is consistent with Stein et al. (2021), who found that evaluations help students feel more engaged and valued by the institution. However, such positive experiences were limited compared to the number of negative experiences reported, with the latter dominating the findings. Students highlighted timing issues, noting that evaluations were often released during busy periods such as examinations. This led to hurried responses or non-participation. These findings are consistent with Vicario et al. (2025), who concluded that timing significantly affects student participation and the quality of responses.

Students also reported confusion resulting from unclear instructions and limited communication regarding the purpose and process of SLE. This aligns with Zhang et al. (2020), who found that a lack of orientation on evaluation tools often results in incomplete or inaccurate feedback. In addition, the study revealed that some students feared victimisation. Although the evaluation is anonymous, students believed negative remarks could somehow be traced back to them. This supports Cantarero and Białek (2026), who noted that fear of retaliation can compromise the honesty of feedback.

Another common negative experience was the perceived lack of action taken after evaluations. Students noted that issues raised repeatedly in previous evaluations were not addressed. This aligns with Deeley et al. (2019), who argue that when institutions fail to close the feedback loop, student trust decreases, leading to disengagement from the evaluation process. Some students also expressed concerns about unfairness and inconsistency in how lecturers were evaluated. This resembles the findings of Esarey and Valdes (2020), who noted that when evaluation criteria are not standardised across departments, students perceive the system as biased and unreliable.

Students collectively envisioned transforming the SLE system into a reliable, transparent and impactful tool for quality improvement. Their recommendations suggest that while students recognise the value of evaluations, the current system requires structural and operational improvements to enhance its credibility, accessibility and effectiveness. A key recommendation centred on the need for stronger policy enforcement and accountability. Students emphasised that for the SLE to be meaningful, institutions must ensure that feedback leads to tangible actions, particularly in cases where lecturers consistently underperform. They suggested linking evaluations to lecturer performance reviews, professional development, or contract renewals. This reflects a desire for the evaluation to move beyond a symbolic exercise towards a formal quality assurance tool.

These views align with findings by Serrano, Miranda Gonzalez and Mourato (2026) and Sheikh et al. (2022), who argue that evaluations contribute most effectively to quality improvement when institutions embed them in performance management policies. Ardill (2025) further emphasises that transparency and lecturer responsiveness to feedback strengthen student trust and reinforce the legitimacy of evaluation processes. The emphasis on visible action mirrors global best practices, demonstrating that accountability and public responsiveness are essential for enhancing the credibility of SLEs.

Students also highlighted significant logistical and technological barriers that hinder effective participation. Many pointed out that the online evaluation system is not sufficiently mobile-friendly, despite smartphones being the primary device used by most students. Others reported challenges related to poor internet connectivity, which result in incomplete evaluations or non-participation. They proposed system upgrades to make the platform more user-friendly and accessible in low-bandwidth environments, as well as the provision of offline alternatives. These concerns are consistent with Fang, Li and Wu (2023) and Ma et al. (2024), who assert that user-friendly, mobile-responsive platforms significantly improve student engagement and response quality. Sanni et al. (2025) also note that offering multiple options, including offline formats, is particularly important in contexts with unstable network access. Compared to studies conducted in technologically advanced settings, the present findings show a greater need for flexible systems that accommodate the infrastructural limitations common in the region.

Improved communication and awareness emerged as another important recommendation. Students felt that inadequate communication regarding evaluation timelines and processes contributed to low participation rates. They proposed structured awareness activities, such as incorporating SLE orientation into first-year induction, providing clear explanations of the purpose and importance of such evaluations, and sending timely reminders before deadlines. These recommendations align with Gan, An and Liu (2021), who highlight that students are more likely to provide thoughtful, meaningful feedback when they understand how their input contributes to teaching improvement. Park (2025) further supports the role of orientation and communication strategies in strengthening a culture of evaluation and improving participation rates. The literature consistently demonstrates that timely reminders improve response rates (Lane & Meth 2021), indicating that the concerns raised in this study are not unique but reflect broader challenges in ensuring active student engagement.

### Limitations and recommendations for future research

This study was restricted to one higher education institution, limiting the generalisability of findings to other settings with different evaluation systems. The study relied solely on student self-reports, which may reflect subjective perceptions rather than objective institutional practices. The absence of lecturer or administrative perspectives also limits the comprehensiveness of the findings. More senior students were more willing to participate in the study than other junior cohorts, which may imply that they were misrepresentation of participants across cohorts, which may lead to bias. Nonetheless, triangulation with existing literature enhanced the trustworthiness of the results. Future studies should broaden the scope by including students from multiple campuses or institutions to enhance generalisability. Mixed-methods research involving both qualitative and quantitative data would increase the depth of analysis. Including lecturers, administrators and policymakers would offer a more holistic understanding of the evaluation process. Longitudinal studies could also examine how implemented recommendations shape teaching and learning outcomes over time.

### Recommendations

Based on the findings, the following recommendations were made: Study participants recommended that the UNAM management should strengthen policy and accountability by enforcing clear policies that ensure that SLE results lead to concrete actions. This includes integrating SLE outcomes into lecturer performance reviews, requiring lecturers to respond to feedback and publicly reporting follow-up actions to enhance accountability and trust. Study participants recommended that the Information Communication and Technology (ICT) department should upgrade the evaluation system to be mobile-friendly, faster and accessible even under poor connectivity conditions. Faculty administrators should ensure well-planned evaluation schedules, timely communication and clear instructions to reduce administrative barriers and increase student participation. Participants strongly suggested that orientation sessions, awareness campaigns and timely reminders should be implemented to educate students on the purpose and importance of evaluations. Lecturers should reassure students regarding confidentiality and promote a safe feedback culture. Improved communication will increase participation and ensure more meaningful, constructive feedback.

## Conclusion

The study concluded that SLEs are recognised by students as essential tools for enhancing teaching effectiveness and promoting accountability within the learning environment. Students perceive SLEs as structured avenues for expressing their learning experiences, identifying teaching strengths and weaknesses and contributing to the improvement of instructional quality. However, the findings show that the effectiveness of SLEs is influenced by several factors. Students demonstrated a clear understanding of the purpose of SLEs, but their participation was strongly shaped by their perceptions of whether the evaluations led to actual change. Motivation to participate increased when feedback resulted in visible improvements; however, many students felt discouraged by the absence of follow-up, limited transparency and perceived institutional indifference. Challenges such as weak communication, poorly timed evaluation periods, inconsistent implementation, fear of victimisation and the limited accessibility of the evaluation system hindered meaningful engagement. Despite these challenges, students acknowledged several benefits of SLEs, including improved lecturer–student relationships, better teaching strategies and enhanced learning environments when evaluations were taken seriously. Overall, the findings reveal that SLEs can contribute significantly to teaching and learning improvement if institutions ensure that the process is transparent, inclusive, technologically accessible and supported by clear policies that hold stakeholders accountable. Students emphasised the need for strengthened communication, accountability and system-level reforms to make the evaluation process more impactful and trustworthy.
